# Case of Monstrosity

**Published:** 1876-08

**Authors:** C. Paul Simon

**Affiliations:** Chicago


### CASE OF MONSTROSITY.

By Dr. C. PAUL SIMON, Chicago.

(Reported to the Chicago Society of Physicians and Surgeons.)

This monstrosity, which I saw with Dr. Landis, sen., of this city, was born illegitimately during the month of April of the present year, in North Chicago, of a woman aged twenty, of German origin, in the seventh month of pregnancy. It belongs to the class of double monsters (Monstres Doubles autositaises, of *Isid. Geoffroy, St. Hilaire*) having a common cord and connected by means of the abdominal walls, and may be called omphalopagus abdominalis. Both were females, facing each other, and apparently of the same development. The connection equalled in thickness the width of the body, and extended from the processus xiphoideus to within a few inches above the pubis, where the cord was attached on one side. The one died shortly after birth, but other lived for about thirty-six hours, her bowels moving in the meantime. A difference of temperature could be detected with the hand immediately on both sides of what should be the common linea alba. No trial of division, and later no examination after death, were allowed.

This case is analogous to that of the Siamese twins, where the cord was common and the connection occurred at the place of the processus xiphoideus. (See Transactions of the College of Physicians of Philadelphia, Phil., 1875.)

